# An Effective and Efficient Sample Preparation Method for 2-Methyl-Isoborneol and Geosmin in Fish and Their Analysis by Gas Chromatography-Mass Spectrometry

**DOI:** 10.1155/2021/9980212

**Published:** 2021-05-08

**Authors:** Liang-liang Tian, Feng Han, Essy Kouadio Fodjo, Wenlei Zhai, Xuan-Yun Huang, Cong Kong, Yong-Fu Shi, You-Qiong Cai

**Affiliations:** ^1^Key Laboratory of East China Sea Fishery Resources Exploitation, Ministry of Agriculture and Rural Affairs, East China Sea Fisheries Research Institute, Chinese Academy of Fishery Sciences, Shanghai 200090, China; ^2^Laboratory of Constitution and Reaction of Matter, UFR SSMT, Université Felix Houphouet Boigny, 22 BP 582, Abidjan 22, Côte d'Ivoire; ^3^Beijing Research Center for Agricultural Standards and Testing, No. 9 Middle Road of Shuguanghuayuan, Haidian District, Beijing 100097, China

## Abstract

The intensive aquaculture strategy and recirculating aquaculture system often lead to the production of off-flavor compounds such as 2-methyl-isoborneol (2-MIB) and Geosmin (GSM). The regular purge and trap extraction followed by analysis with gas chromatography-mass spectrometry (GC-MS) usually involve a complicated assembly of facilities, more working space, long sample preparation time, and headspace solid-phase microextraction (SPME). In this work, a method with easier sample preparation, fewer and simplified facilities, and without SPME on GC-MS analysis is developed for the determination of 2-MIB and GSM in fish samples. Unlike previous methods, solvent extract from samples, QuEChERS-based cleanup, and solid-phase extraction for concentration are applied. The LOD (S/N > 3) and LOQ (S/N > 10) of this method were validated at 0.6 *μ*g/kg and 1.0 *μ*g/kg for both 2-MIB and GSM, which are under the sensory limit (1 *μ*g/kg). Application of this method for incurred fish samples demonstrated acceptable analytical performance. This method is suitable for large-scale determination of 2-MIB and GSM in fish samples, owing to the use of simple facility and easy-to-operate procedure, rapid sample preparation, and shorter time for GC-MS analysis without SPME.

## 1. Introduction

The continuous increase in aquaculture production provides more than 50% of aquatic product for global demand. To support the nutrition and food security around the world, the intensive aquaculture strategy is adopted in many farm areas [1]. In parallel, the recirculating aquaculture system (RAS) is also developed to avoid the contamination of water system, and to better control the environment [2] for farming and to save water [3]. However, the intensive farming and RAS [4, 5] can result in the occurrence of off-flavor compounds and the tainting of fishery product, which adversely impact the fish-farming industry [6]. The earthy-musty off-flavor tainting in the fish product can lead to complaints from consumers and the decrease of its market value and to be perceived as unfit for retail [7]. Removal of the off-flavor in fishery product leads to profit reduction [8].

One of the most popular sources of off-flavors originates from the secondary metabolic products in the environmental microbial, such as cyanobacteria [9], actinomycetes [10, 11], or fungi [12]. The dominant objectionable compounds are identified as 2-methyl-isoberneol (2-MIB) and geosmin (GSM), which are responsible for the earthy-musty off-flavor in water or fish product [13]. Usually, only trace amounts of the two taste and odor compounds persist in fish flesh. However, the sensory thresholds for human organoleptic response are quite sensitive and are reported to be no more than 1 *µ*g/kg in different fishes [14]. This tiny residue can lead to unpalatable taste for consumers, decreasing the quality of fish product, and leading to subsequent loss for farmers. There are instruments or methods based on organoleptic test for the evaluation of the tainting status of off-flavors in fish [15]. A panel of trained staffs needs to be prepared for complicated and time-consuming assessment and scores [16]. The sensory threshold varies, depending on the flavors of fish [17]. Moreover, the off-flavors can only be assessed without the identification of their cause and quantification [18]. To determine the exact origin and extend of off-flavor by these two most common compounds, methods for their determination in fish are reasonably required [19].

Many methods have been developed for the determination of 2-MIB and GSM in water environments over the past 30 years [20]. Relatively fewer methods are reported for their determination in fish flesh. The prevalent methods are carried out on gas chromatography-mass spectrometer, regardless of the water or fish flesh. The extraction [10] from matrix often involves the purge and trap process [21], with many different assisted distillation and extraction techniques [22]. Moreover, the trapped residues usually undergo enrichment before GC-MS analysis [23] such as solid-phase microextraction [24]. When it comes to the large-scale determination of samples, the purge and trap process involve assembly of complicated devices and require more working space for parallel operation. Furthermore, it requires large sampling weight for these methods, and the enrichment [25]. The headspace sampling for concentrating analytes from fish matrix demands extra time for recleaning for the next sample analysis. Besides, owing to the semivolatile properties of 2-MIB and GSM, the headspace sampling [26] or purge-trap process can lead to a poor reproducibility for repeated analysis [27]. Therefore, these methods are labor-intensive and time-consuming and rely on more consumables, facilities, and spaces for large-scale sample analysis.

**Figure 1:**
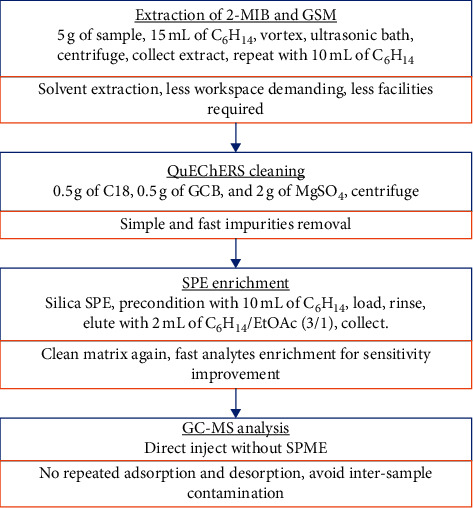
Workflow for analysis of 2-MIB and GSM residues in the fish samples.

In this study, we aimed to develop a method for large-scale determination of 2-MIB and GSM, with simple solvent extraction, cleaning, enrichment, and sampling process, as well as fewer facilities usage and easier operation, as shown in Figure 1. To overcome the shortages by purge and trap extraction process, a liquid extraction from fish flesh matrix is investigated. QuEChERS method, as a popular and rapid sample extract cleaning method in pesticide and veterinary detection, has been examined for the cleaning of the extract in this work. SPE method is applied for the enrichment of analytes and solvent replacement. GC-MS analysis of these two compounds is performed without SPME for concentration. The performance of this method is evaluated on the limit of detection, recovery, and linear range. Furthermore, it is validated with incurred samples.

## 2. Materials and Methods

### 2.1. Reagents and Materials

*N*-hexane (HPLC grade), ethyl acetate ester, and acetone were supplied by J. T. Baker. 2-Methyl-isoborneol (100 *μ*g/mL, purity: >98%) and geosmin (100 *μ*g/mL, purity: >98%) were obtained from Dr. Ehrenstorfer. They were diluted to 1 *μ*g/mL using *n*-hexane and stored at −18°C in the dark before use. Sodium sulfate anhydrous and magnesium sulfate anhydrous were provided by Aladdin Industrial Corporation. Solid-phase extract cartridge (Silica 500 mg/3 mL) and dispersive solid-phase extract (C18, graphitized black carbon, PSA) were purchased from Agela Technologies. Grass carp and crucian carp were bought from local farm produce market in Shanghai.

### 2.2. Instrument

Gas chromatography coupled to mass spectrometer (TSQ QUANTUM GC, Thermofisher Scientific) was used for GC-MS analysis. Balance (0.01 g, sartorius company, Germany), solid-phase extraction device (Supelco company), and ultrasonic cleaner (Branson) were also used during the experiment.

### 2.3. QuEChERS Materials Adsorption Profile Test

0.5 g of graphitized black carbon, C18, PSA, sodium sulfate anhydrous, silica, alumina (neutral or alkaline), florisil, chitosan, and diatomite were added to 5 mL of these solutions, respectively. The solutions were stirred and sat quiet to collect the supernatant, which was prepared for GC-MS detection. Each adsorbent was examined with 2 replicates.

### 2.4. Extraction and Enrichment of 2-MIB and GSM from Fish

15 mL of *n*-hexane was added into a plastic centrifuge tube containing 5*g* of sample, which was sealed immediately to avoid volatile loss. This mixture was vortexed for 5 min at 2500 rpm and treated with ultrasonic bath for 15 min. To get the supernatant collected, the sample was centrifuged at 5000 *×*g for 8 min. The remaining sample was extracted with 10 mL *n*-hexane again. The extract was combined in a 50 mL plastic centrifuge tube. This extract was mixed with 0.5*g* of C18 adsorbent, 0.5*g* of graphitized black carbon, and 2*g* of magnesium sulfate anhydrous and was vortexed for 7 min, at 2500 rpm, followed by centrifugation at 1400 *×*g for 5 min to obtain the supernatant. The silica SPE cartridge was preconditioned with 10 mL *n*-hexane before the passthrough of extract, which was rinsed with 3 mL *n*-hexane after loading, and dried by vacuum. The analyte was eluted with 2 mL of *n*-hexane/ethyl acetate (3/1), and the cartridge was blown to dry during eluting. The eluate was then collected in a vial and vortexed for GC-MS analysis.

### 2.5. GC-MS Determination

The HP-5 MS capillary column (30 m × 0. 25 mm × 0. 5 *μ*m) was used for GC separation with constant nitrogen gas flow at 1.0 mL/min. The programmable temperature procedure was initially set at 60°C for 1 min, increased to 120°C at the rate of 5°C/min, and kept for 3 min. Then, it was increased to 170°C at the rate of 5°C/min and kept for 1 min. Subsequently, it went to 280°C with 20°C/min and was kept for 5 min. 1 *μ*L of the sample solution was injected into the sampling port at 250°C without diverting.

In the mass spectrometry analysis, electron ionization (EI) source was used with a source temperature at 250°C, MS transfer line temperature at 280°C, and electron energy at 70 eV. Selective ion monitoring (SIM) was used for data acquisition. The quantitative ion for 2-MIB is m/z 94.9, and its qualitative ions are m/z 94.9, 107.9, and 134.9, while the quantitative ion for GSM is m/z 111.9, and its qualitative ions are m/z 111.9, 124.9, and 96.9.

### 2.6. Real Incurred Samples Preparation

Crucian carp and grass carp were used for incurred sample preparation. Before incurring, 5 crucian carp (ca. 500 g/each) and 5 grass carp (1500 g/each) were adaptively cultured in a 300-liter tank for 3 days with regular feeding and bubbling. Half of the water was replaced every day during culture. For incurring with 2-MIB and GSM, 300 liters of water was prepared by spiking 100 *μ*g/L of the two contaminants. And then, the fishes were bathed for 8 hours in the contaminated water before transferring to clean water. After depuration in clean water for 2 hours, the incurred fish was sampled immediately by cutting off the fillets and storing in refrigerator.

## 3. Results and Discussion

### 3.1. Adsorption of Different QuEChERS Materials

**Figure 2:**
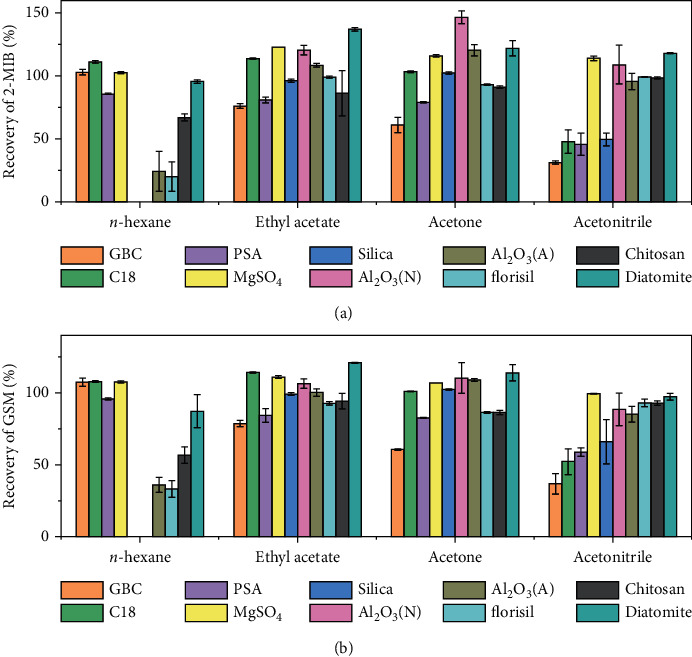
Recoveries of 2-MIB (a) and GSM (b) after adsorption in different solvents with different adsorption materials. Spiked concentration: 50 ng/mL.

In the beginning, to use the QuEChERS materials for cleanup of the extract, the adsorption profiles of different materials under various solvents at the concentration of 50 ng/mL of 2-MIB and GSM in *n*-hexane, ethyl acetate, acetone, and acetonitrile are examined. These solvents are intended to extract 2-MIB and GSM from flesh samples. Results of the recoveries of the analytes after adsorption by different adsorbents in different solvents for 2-MIB and GSM are shown in Figure 2. In the solvent of *n*-hexane, C18, graphitized black carbon and magnesium sulfate show less than 10% loss of the two analytes, while PSA, alumina (alkaline), florisil, diatomite, and chitosan show 10–80% adsorption of these analytes. In contrast, the alumina (neutral) and silica adsorb all the analytes. In the solvent of ethyl acetate, graphitized black carbon, magnesium sulfate anhydrous, silica, alumina (neutral or alkaline), florisil, and chitosan do not adsorb the analytes. C18 and PSA adsorbed 10–40% of these analytes. In acetone, C18 and PSA also adsorb 10–40% of the analytes and other materials show no significant adsorption. In acetonitrile, except magnesium sulfate anhydrous, diatomite, florisil, and chitosan, other materials show around 10–70% adsorption for both analytes.

The above four solvents can dissolve various disturbing components when used to extract analytes from fish flesh. The combined use of these materials was preferred for better cleaning of these extracts. Besides, we would like to do analyte enrichment after cleanup. It is noticed in Figure 2 that silica can be an adsorbent for enrichment when analytes are dissolved in *n*-hexane. *N*-hexane is chosen as it can extract the analytes with good solubility [4, 11], undergo QuEChERS cleanup with low analytes loss, and promise feasibility for following concentration process.

Therefore, we checked the combined use of QuEChERS materials that shows less than 10% adsorption of analytes in *n*-hexane. Among these material combinations, 0.5 g of C18, 0.5 g of graphitized black carbon, and 2 g of magnesium sulfate anhydrous can keep more than 95% recovery after QuEChERS cleaning. Therefore, they are chosen as QuEChERS materials for extract cleaning. Furthermore, the performance of *n*-hexane as an extract solvent for the analytes is also examined as shown in Figure S1 and Table S1 (see Supplementary Materials).

### 3.2. Concentrate of Analytes

To increase the sensitivity of detection with small sampling weight, the concentration process is necessary and expected to be most feasible. According to Figure 2, 2-MIB and GSM can be adsorbed by silica when dissolved in *n*-hexane and have no adsorption when dissolved in ethyl acetate and acetone. Therefore, the extract of the two analytes with a large volume of *n*-hexane can be enriched on silica cartridge and can further be eluted with small volume of solvent mixture. The adsorption rate of these two analytes is examined with silica cartridge (500 mg, 3 mL). The result shows no residue in the collected solvent, indicating the complete adsorption (100%) of 2-MIB and GSM on the silica cartridge.

**Figure 3:**
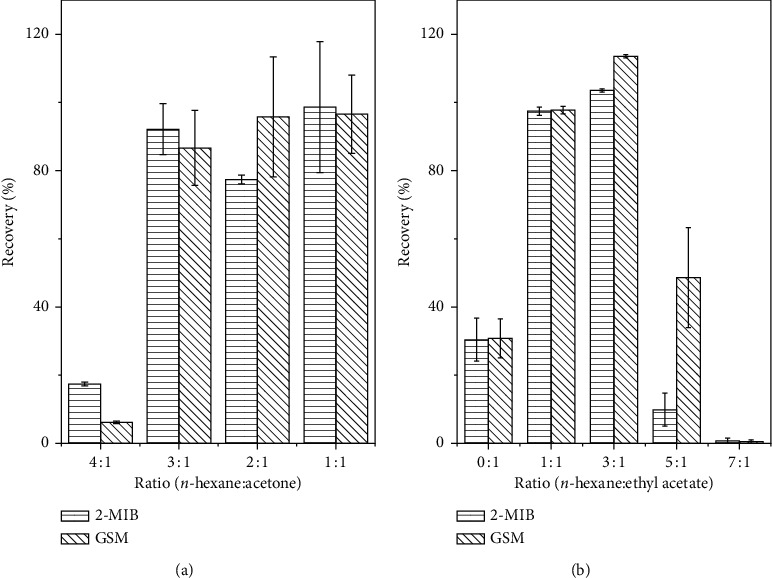
Recovery of 2-MIB and GSM with *n*-hexane and acetone mixture (a) or *n*-hexane and ethyl acetate mixture (b) as elute on silica cartridge. Spiked concentration: 100 ng/mL.

Furthermore, the elution solvent for the release of adsorbed analytes from the silica cartridge was investigated. After washing with *n*-hexane, the loading cartridge was dried under nitrogen blow and eluted with different solvent mixture. Initially, the solvent mixture with different ratio of *n*-hexane and acetone was examined. As displayed in Figure 3, high percentage of acetone can increase the elution rate on the cartridge. However, the stability of the eluted analytes is quite low, with an obvious difference between 2-MIB and GSM. Under the ratio of 1 : 1 between *n*-hexane and acetone, the elution rate can reach around 100%. To find a highly efficient elute solvent, the solvent mixture with different ratio of *n*-hexane and ethyl acetate was further examined. As shown in Figure 3, the pure ethyl acetate does not produce higher elution rate, while the ratio of 1 : 1 and 3 : 1 between *n*-hexane and ethyl acetate can elute almost 100% of 2-MIB and GSM. However, further increase in the ratio of *n*-hexane leads to the decrease of elution rate and even results in no elution at all. When applied in real samples, it should be noted that there is more impurity peak on the chromatogram for 2-MIB and GSM in the collected elute with the mixture of *n*-hexane and acetone than with the mixture of *n*-hexane and ethyl acetate as displayed in Figure S6 (Supplementary Materials). Finally, the mixture of *n*-hexane and ethyl acetate (3/1) is selected as an ideal elute solvent with high elution rate, less impurity peak on the chromatogram, and good stability.

### 3.3. Matrix Effect

**Figure 4:**
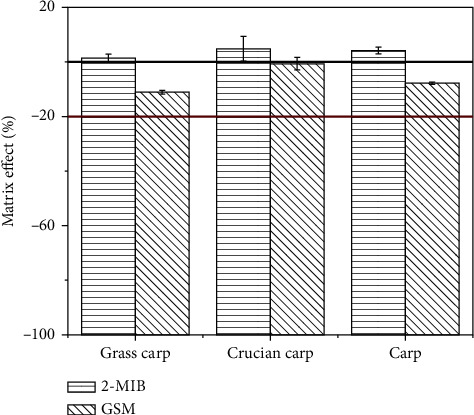
Matrix effect of 2-MIB and GSM in the reconstitution solution obtained from grass carp, crucian carp, and carp undergoing extraction and cleaning procedure.

The matrix effect of this method was evaluated with grass carp, crucian carp, and carp samples as they have been frequently reported to accumulate these two off-flavors [9]. For this purpose, 100 ng of 2-MIB and GSM were spiked in 1 mL of the blank matrix solution, which were obtained by extracting from the fish samples, followed by cleaning with QuEChERS method and eluting with silica cartridge. The matrix effect was calculated through the ratio between the response areas of analytes in the spiked solution and in the solvent at the same concentration, which is further reduced by one and is multiplied by 100%. As displayed in Figure 4, the matrix effect for 2-MIB is no more than 5% in the three matrices, and for GSM, the matrix effect is the weakest in crucian carp at 1%∼3% and the strongest in grass carp at 11%. According to these results, the matrix effect for the two analytes in all these matrices is acceptable for calibration with solvent standards and quantification with no apparent bias.

### 3.4. Method Validation

After optimization of several parameters of the method, its performance was validated with linearity, LOD, LOQ, and recovery. The result shows linearity for standard solutions of 6 different concentrations of 2–500 ng/mL with a relative coefficient of 0.9997 for both 2-MIB and GSM. The LOD is confirmed with grass carp at the concentration when the ratio of signal to noise ≥3 and LOQ is confirmed when the ratio of signal to noise ≥10, with the accuracy of 70–120% and an imprecision <20%. As a result, both 2-MIB and GSM are confirmed with LOD at 0.6 *μ*g/kg, and LOQ of both at 1.0 *μ*g/kg, which are below the sensory limit of humans, indicating the practicability for 2-MIB and GSM detection.

The recoveries were obtained in grass carp and crucian carp with 2.0, 10.0, and 20.0 *μ*g/kg of 2-MIB and GSM spiked in the samples.  Table S3 and Table S4 (Supplementary Materials) show the results of recoveries at these spiking levels. All the average recoveries for these different spiking levels are higher than 80% with a relative standard deviation of no more than 8% in both grass carp and crucian carp. These results indicate good recoveries and stability of the method for detecting 2-MIB and GSMin fish samples.

**Figure 5:**
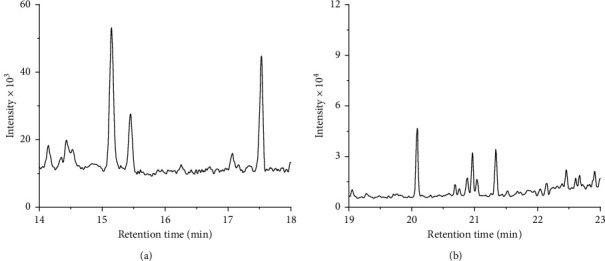
Typical chromatograms of 2-MIB (a) and GSM (b) in incurred grass carp after sample preparation (retention time: 2-MIB, 15.15 min, GSM, 21.33 min).

To further test the practicability of the method, the real incurred sample, grass carp, and a liver sample of grass carp were analyzed for their content of these two analytes. These samples show the presence of off-flavor compounds through organoleptic evaluation. As shown in Table 1, one of the grass carp has a residue of 2-MIB of about 10 times the sensory limit and a residue of 3 times the sensory limit of GSM. The other grass carp accumulates a much higher amount of 2-MIB and GSM, which are around 40 and 15 times the other grass carp. In the liver sample, residues of 2-MIB and GSM are found, which are much higher than their residue in flesh samples. A typical chromatogram of 2-MIB and GSM in grass carp 1 is displayed in Figure 5. This result demonstrates good separation of analytes and interference for real samples. The relative RSDs for flesh samples are less than 5%, indicating good stability in these matrices. However, the detected residue in the liver varies even in the same sample. It can be attributed to the less sampling amount that results in the poor homogeneity of samples and volatile loss during sample preparation. The above results further demonstrate the feasibility of this method for 2-MIB and GSM determination in off-flavor fish samples.

## 4. Conclusions

In general, a method for large-scale determination of 2-MIB and GSM in fish samples has been developed through this work. The extraction mechanism, enrichment, and injection mode are different from the previous reported purge and trap method [8, 21]. The method allows straightforward liquid extract from fish flesh instead of purge and trap extraction, followed by cleanup with optimized QuEChERS material and further concentration with silica cartridge. A sampling process without SPME has further simplified the GC-MS analysis process. The performance of this method is evaluated and validated with incurred samples, showing its practicability. The LOD of this method is under the sensory limit of fish samples. Besides, this method involves no complicated facility and easy-to-operate procedure and rapid sample preparation. It allows large-scale determination of 2-MIB and GSM in fish samples and can also find extended application in other semivolatile compound determination.

## Figures and Tables

**Table 1:** Results of the 2-MIB and GSM residues in the incurred samples.

Sample	2-MIB (*μ*g/kg)	Average (*μ*g/kg)	RSD (%)	GSM (*μ*g/kg)	Average (*μ*g/kg)	RSD (%)
Flesh 1	11.7/11.8/12.2	11.9	2.22	3.43/3.15/3.19	3.26	4.65
Flesh 2	441.5/507.5/358.4	435.8	17.1	45.3/56.0/40.2	47.2	17.1
Liver	704.6/781.2/1063	849.6	22.2	224.9/253.2/318.8	265.6	18.1

## Data Availability

The data used to support the findings of this study are available from the corresponding author upon request.

## Supplementary Materials

Supplementary Materials The following materials are included in the supplementary materials. Fragment ions identification, optimization of GC-MS parameters, extraction of analytes, concentrate with evaporation, optimization on the rinsing solvent, and examination on the silica in the cartridge. Figure S1: full scan mass spectra and chemical structures. Figure S2: effect of sampling port temperature. Figure S3: signal of the two analytes under different initial temperature during GC separation. Figure S4: signal of the two analytes under different temperature ramp rate during GC separation. Figure S5: chromatography profile of the two analytes extracted from carp and crucian carp with *n*-hexane and acetonitrile. Table S1: recovery of the two analytes with acetonitrile and *n*-hexane as extract solvents. Table S2: recovery of the two analytes under different concentrate mode. Figure S6: chromatograms of the two analytes elution from silica cartridge. Figure S7: recoveries of the two analytes in rinsing solution when rinsed with different mixture of *n*-hexane and ethyl acetate on the silica cartridge. Figure S8: recoveries of the two analytes on different silica cartridges in the practical spiked extract. Table S3: recoveries under different spiking levels in grass carp. Table S4: recoveries under different spiking levels in crucian carp.
