# Supplementary material for: An Effective and Efficient Sample Preparation Method for 2-Methyl-Isoborneol and Geosmin in Fish and Their Analysis by Gas Chromatography-Mass Spectrometry
Source: Int J Anal Chem. 2021 May 8;2021:9980212. doi: 10.1155/2021/9980212 (PMC8128619; doi:10.1155/2021/9980212)
Supplement: Supplementary Materials — The following materials are included in the supplementary materials. Fragment ions identification, optimization of GC-MS parameters, extraction of analytes, concentrate with evaporation, optimization on the rinsing solvent, and examination on the silica in the cartridge. Figure S1: full scan mass spectra and chemical structures. Figure S2: effect of sampling port temperature. Figure S3: signal of the two analytes under different initial temperature during GC separation. Figure S4: signal of the two analytes under different temperature ramp rate during GC separation. Figure S5: chromatography profile of the two analytes extracted from carp and crucian carp with n-hexane and acetonitrile. Table S1: recovery of the two analytes with acetonitrile and n-hexane as extract solvents. Table S2: recovery of the two analytes under different concentrate mode. Figure S6: chromatograms of the two analytes elution from silica cartridge. Figure S7: recoveries of the two analytes in rinsing solution when rinsed with different mixture of n-hexane and ethyl acetate on the silica cartridge. Figure S8: recoveries of the two analytes on different silica cartridges in the practical spiked extract. Table S3: recoveries under different spiking levels in grass carp. Table S4: recoveries under different spiking levels in crucian carp. [file 9980212.f1.docx]

# International Journal of Analytical Chemistry

# An effective and efficient sample preparation method for 2-methyl-isoborneol and geosmin in fish and their analysis by gas chromatography-mass spectrometry

Liang-liang Tian,^1^ Feng Han,^1^ Essy Kouadio Fodjo,^3^ Wenlei Zhai,^2^ Xuan-yun Huang,^1^ Cong Kong,^1^ Yong-fu Shi,^1^ You-qiong Cai^1^

^1^ Key Laboratory of East China Sea Fishery Resources Exploitation, Ministry of Agriculture and Rural Affairs, East China Sea Fisheries Research Institute, Chinese Academy of Fishery Sciences, Shanghai 200090, P. R. China.
^2^ Beijing Research Center for Agricultural Standards and Testing, No. 9 Middle Road of Shuguanghuayuan, Haidian District, Beijing 100097, P. R. China.
^3^Laboratory of constitution and reaction of matter, UFR SSMT, Université Felix Houphouet Boigny, 22 BP 582 Abidjan 22, Cote d’Ivoire

Correspondence should be addressed to Cong Kong; kongc@ecsf.ac.cn

**Fragment ions identification**

To find the characteristic ion fragments of 2-MIB and GSM, the standard solution of 2 μg/mL was prepared, and full-scanned with a mass spectrometer. Through the mass database search and the comparison with previous literature report *^1, 2^*, considering the relative abundance of each fragment ion (as displayed in **Figure S**1), the m/z 94.9/107.9/134.9 and 111.9/124.9/96.9 are selected as the characteristic ions for 2-MIB and GSM, respectively.


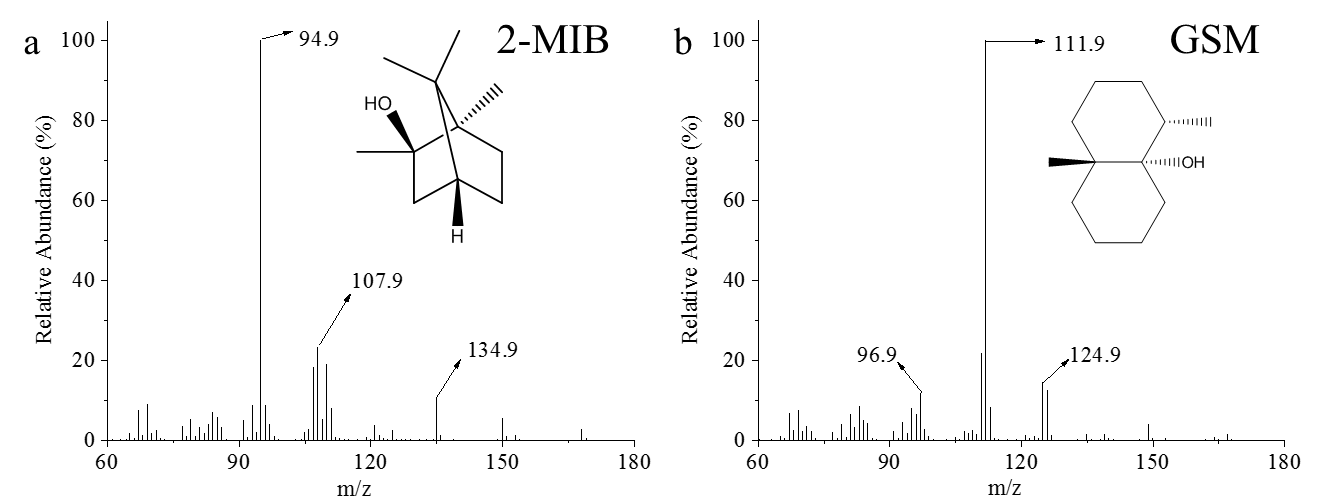


**Figure S**1. Full scan mass spectra and chemical structures of 2-MIB (a) and GSM (b) after EI ionization at 2 μg/mL of the standard analytes.

**Optimization of GC-MS parameters**

The temperature of the sampling port was examined to obtain a suitable parameter*^3, 4^*. Different temperatures at 200, 250 and 280℃ were tested for their effect on the sensitivity of analytes. As displayed in **Figure S**2, the result shows no significant response of signal height and area. However, the signal to noise ratio (S/N) at different sampling port temperature varies (as shown in Figure S2.c), in which the highest S/N is obtained at 250℃ for both 2-MIB and GSM, and selected for the following analysis.


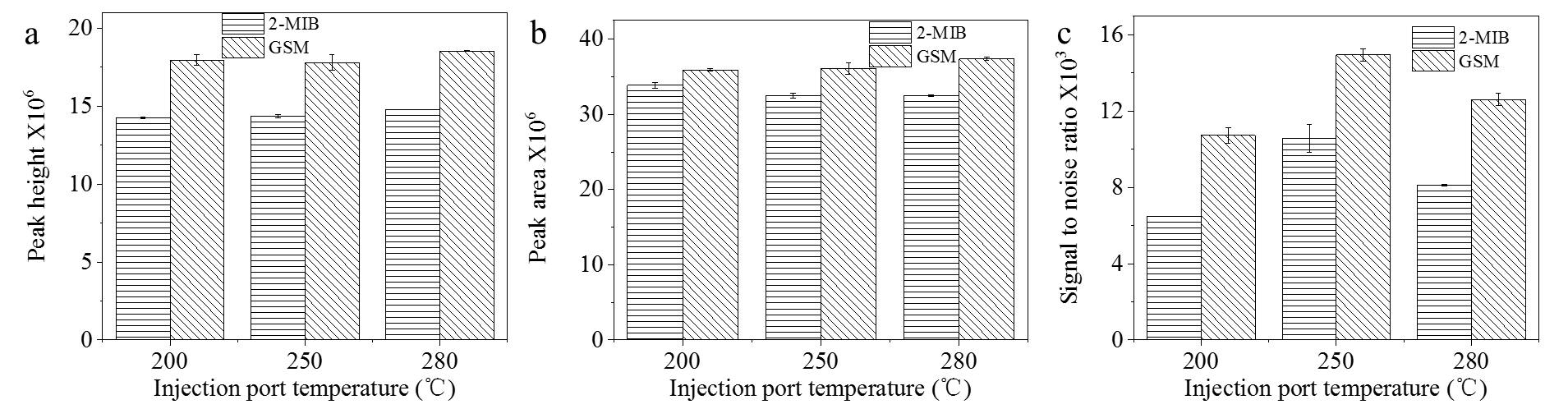


**Figure S**2. Effect of sampling port temperature on the peak height (a), area (b) and signal to noise ratio (c) of 2-MIB and GSM.

The different initial temperatures, namely 60℃ and 100℃ for GC separation were also examined*^4^*. The results show no significant difference in the signal area for analytes between these two temperatures. As shown in **Figure S**3, the peak height and signal to noise ratio was much higher at 60℃ than at 100℃. The lower signal to noise ratio can be attributed to lower peak height and broad peak width at the higher temperature, while the peak area is kept almost constant. Therefore, 60℃ is chosen as the initial temperature for further tests.


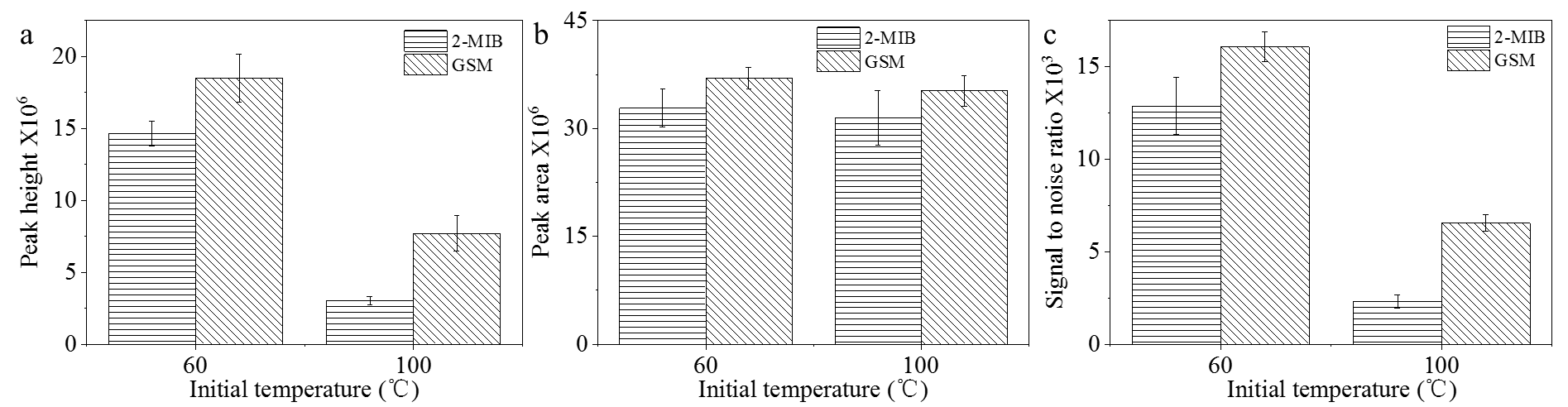


**Figure S**3. Peak height (a), area (b) and signal to noise ratio (c) of the two analytes under different initial temperatures during GC separation.

After that, the ramp rate for temperature increase was investigated at 2℃/min, 5℃/min, 8℃/min, 9℃/min, 10℃/min, 11℃/min, 12℃/min, 13℃/min, 14℃/min, 15℃/min, with standard analytes at the concentration of 100ng/mL. As shown in **Figure S**4, similar to the trend in initial temperature examination, the signal area do not change in parallel with the temperature ramp rate. While the signal height and corresponding signal to noise ratio increased, indicating that the faster ramp rate produces narrower signal peaks and subsequently increase its height. To reach a higher sensitivity, a faster ramp rate would be applied if no interference impurity appeared in the blank sample and around the target peak region for the positive sample.


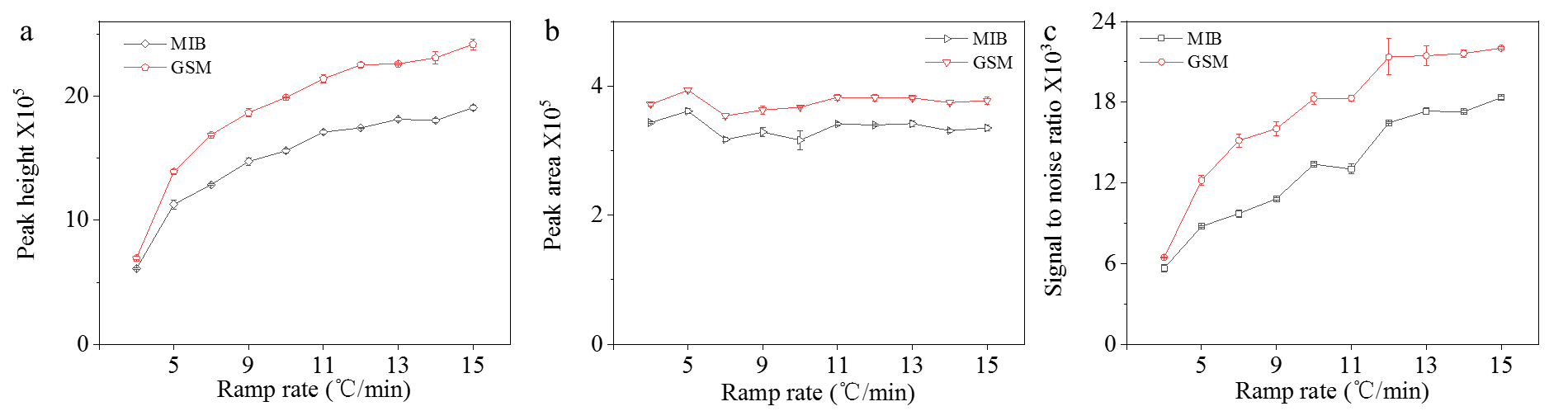


**Figure S**4. Peak height (a), area (b) and signal to noise ratio (c) of the two analytes under different temperature ramp rates during GC separation.

**Extraction of analytes**

The solvent, acetonitrile and n-hexane were individually tested for their efficiency in the extraction of analytes from fish samples. For this purpose, to 5 g of fish samples, 750 ng 2-MIB and GSM were added and mixed. 15 μL of acetonitrile or n-hexane was used for extraction and further stirred at 2500 rpm for 5 min. The extract was collected through centrifugation and added with QuEChERS materials (0.5 g of graphitized black carbon, 0.5 g of C18 adsorbent, 0.5 g of PSA adsorbent, 2 g of magnesium sulfate anhydrous). After adequate stirring, the extract was filtered through a 0.22 μm hydrophobic membrane for GC-MS analysis.

Acetonitrile and n-hexane display different recovery efficiency in the extraction of the 2-MIB and GSM from fish samples. As shown in **Figure S5**, the impurity peaks for the extract of carp or crucian carp by acetonitrile or n-hexane show a noticeable difference. The extract with acetonitrile is cleaner in the chromatography than the extract using n-hexane. More interference peak appears when applying n-hexane for extraction from fish samples, which may significantly decrease the signal to noise ratio for analysis at the concentration close to the limit of detection. As shown in **Table S1**, the recovery ranges between 37-76% when using acetonitrile to extract, and ranges between 53-124% when using n-hexane. These recovery differences can be caused by the different adsorption efficiency for analytes when the extract is mixed with QuEChERS materials. Nonetheless, the QuEChERS materials can also influence the adsorption of impurities, leading to different impurity peaks in the chromatography. Although the chromatogram is cleaner when the acetonitrile is used as extract solvent, considering the recovery and its compatibility for following QuEChERS and SPE concentration step, the n-hexane is preferred to be the extracting solvent for 2-MIB and GSM.


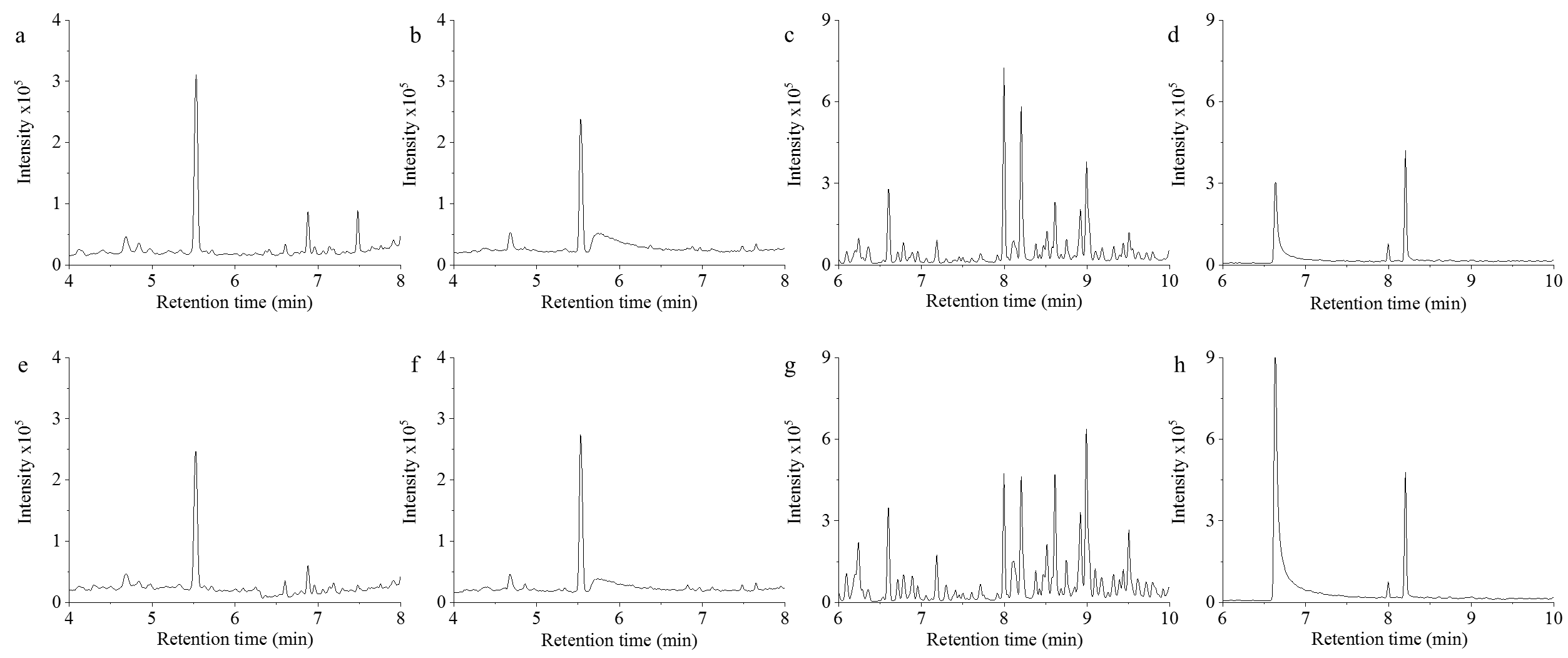


**Figure S**5. Chromatography profile of 2-MIB (a, b, e, f) and GSM (c, d, g, h) when extracted with n-hexane (a, e, c, g) and acetonitrile (b, f, d, h) after spiking in carp (a, b, c, d) and crucian carp (e, f, g, h) (spiked concentration 750 ng/5 g). (Parameters for GC separation was not in optimum as shown in the maintext. Retention time: 2-MIB, 5.53 min, GSM, 8.21 min).

**Table S**1. Recovery of 2-MIB and GSM with acetonitrile and n-hexane as extract solvents.

| Acetonitrile | Recoveries ( %) | | n-hexane | Recoveries ( %) | |
| --- | --- | --- | --- | --- | --- |
|  | 2-MIB | GSM |  | 2-MIB | GSM |
| carp | 37.1~57.0 | 51.7~67.7 | carp | 67.40~77.8 | 53.1~124.2 |
| crucian carp | 66.1~71.7 | 73.3~76.0 | crucian carp | 67.1~83.9 | 91.9~100.4 |

**Concentrate with evaporation**

To concentrate the extract, nitrogen blowing and vacuum evaporation were both examined. 1 mL of 2-MIB and GSM (200 ng/mL) was prepared and concentrated with the two methods. As shown in **Table S**2, a high flow rate of nitrogen without heat bath can only recover less than 3.3% of the two analytes. While the recovery of the analyte can only go up to no more than 32.6% even with a low flowrate without heat bath. Concentrating with vacuum evaporation also does not result in good recovery, with the recovery of less than 33.5% and less than 22.1% are obtained with low (300 mbar) or high (100 mbar) vacuum conditions. Therefore, attempt to concentrate the extract with the two methods are not suitable as they lead to less recovery and stability.

**Table S**2. Recovery of 2-MIB and GSM under different concentrate mode.

| concentrate mode | recovery of 2-MIB  (%) | recovery of GSM  (%) |
| --- | --- | --- |
| high gas flux without heat | 0 | 0~3.3% |
| low gas flux without heat | 2.2%~6.68% | 13.0%~32.6% |
| rotating evaporate under low vacuum | 1.04%~11.4% | 8.6%~33.5% |
| rotating evaporate under high vacuum | 0~10.3% | 0~22.1% |


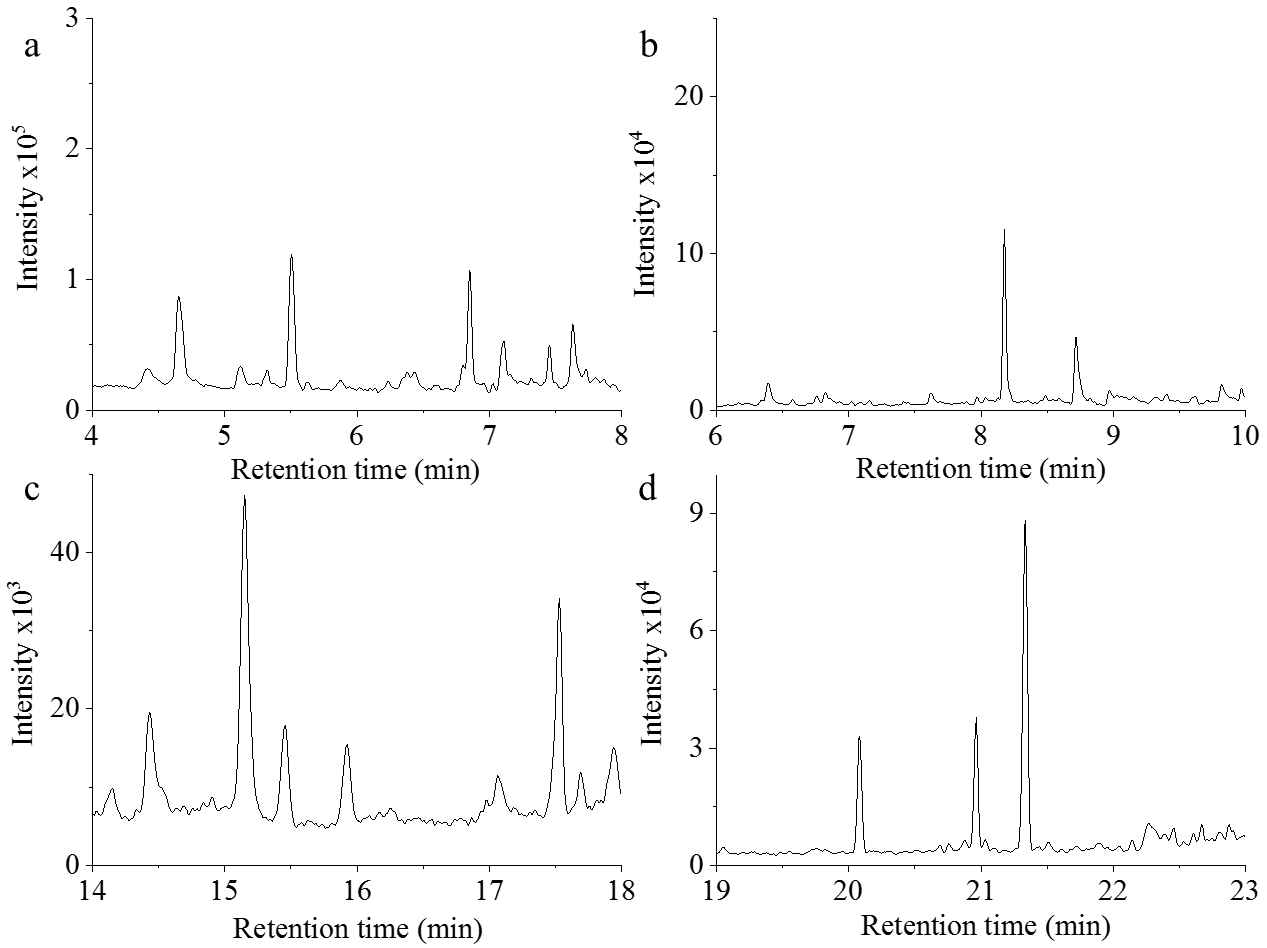


**Figure S**6. Chromatograms of 2-MIB (a) and GSM (b) eluted with n-hexane and acetone, after extracted from the spiked crucian carp, and loaded on silica cartridge (Parameters for GC separation was not in optimum as shown in the main text. Retention time: 2-MIB, 5.53 min, GSM, 8.21 min). The chromatogram of 2-MIB (c) and GSM (d) eluted with n-hexane and ethyl acetate, after extracted from the spiked crucian carp, and loaded on silica cartridge. (Retention time: 2-MIB, 15.15 min, GSM, 21.33 min)

**Optimization on the rinsing solvent**

To keep the loaded analytes on the silica cartridge and clean off more impurities, the rinsing solvent was tested with different ratios of n-hexane and ethyl acetate. 100 ng of dissolved 2-MIB and GSM in the blank matrix solution (Crucian carp) were loaded on the cartridge after conditioning with n-hexane, the rinsing solvent was collected after passing through the cartridge, and the detection of these residues was performed with GC-MS. As displayed in **Figure S**7, the ratio of ethyl acetate can prominently influence the rinsing effect, where almost 100 % of the loaded analytes are eluted with the ratio of 10:1, and slight residues in the rinsing solvent with the ratio of 20:1. The ratio of 30:1 between n-hexane and ethyl acetate can keep the analyte on the cartridge. However, this ratio is quite close to pure n-hexane and the ratio of 20:1 can elute analyte when the ratio is not well controlled. Therefore, n-hexane is finally selected as the rinsing solvent mixture to clean the analytes, and avoid analytes loss.

**Figure S**7. Recoveries of 2-MIB and GSM in rinsing solution when rinsed with different mixture of n-hexane and ethyl acetate on the silica cartridge.

**Examination on the silica in the cartridge**

The amount of silica inside the cartridge can influence the adsorption and recoveries for enrichment and clean-up of practical samples. The small amount of loading material can save solvent for eluting and increase the concentrating factor, while the adsorption rate can not be guaranteed. Therefore, two different amounts of silica material in the cartridge, 200 mg/3 mL and 500 mg/3 mL were tested for clean-up and recoveries of the two analytes in the practical matrix.

The real samples, grass carp and crucian carp, were extracted with n-hexane as matrix blank and spiked with 100 ng of 2-MIB and GSM. The extract was loaded, on Silica cartridges (200 mg/3 mL and 500 mg/3 mL), rinsed with the n-hexane and eluted with the mixture of n-hexane and ethyl acetate **(3/1)**, respectively. The elute was determined with GC-MS to examine the recoveries of analytes. As displayed in **Figure S**8, the recoveries for 2-MIB are not significantly different on the two cartridges, while for GSM, the cartridge (200 mg/3mL) recovered 60%, which is 110% when the cartridge (500 mg/3mL) is applied. This can be attributed to the decreased loading efficiency for GSM when less loading material is applied in the cartridge and further results in the loss of GSM with loading solvent and reduces recoveries in the elute. Therefore, the 500 mg/3mL is selected as enrichment cartridge finally.


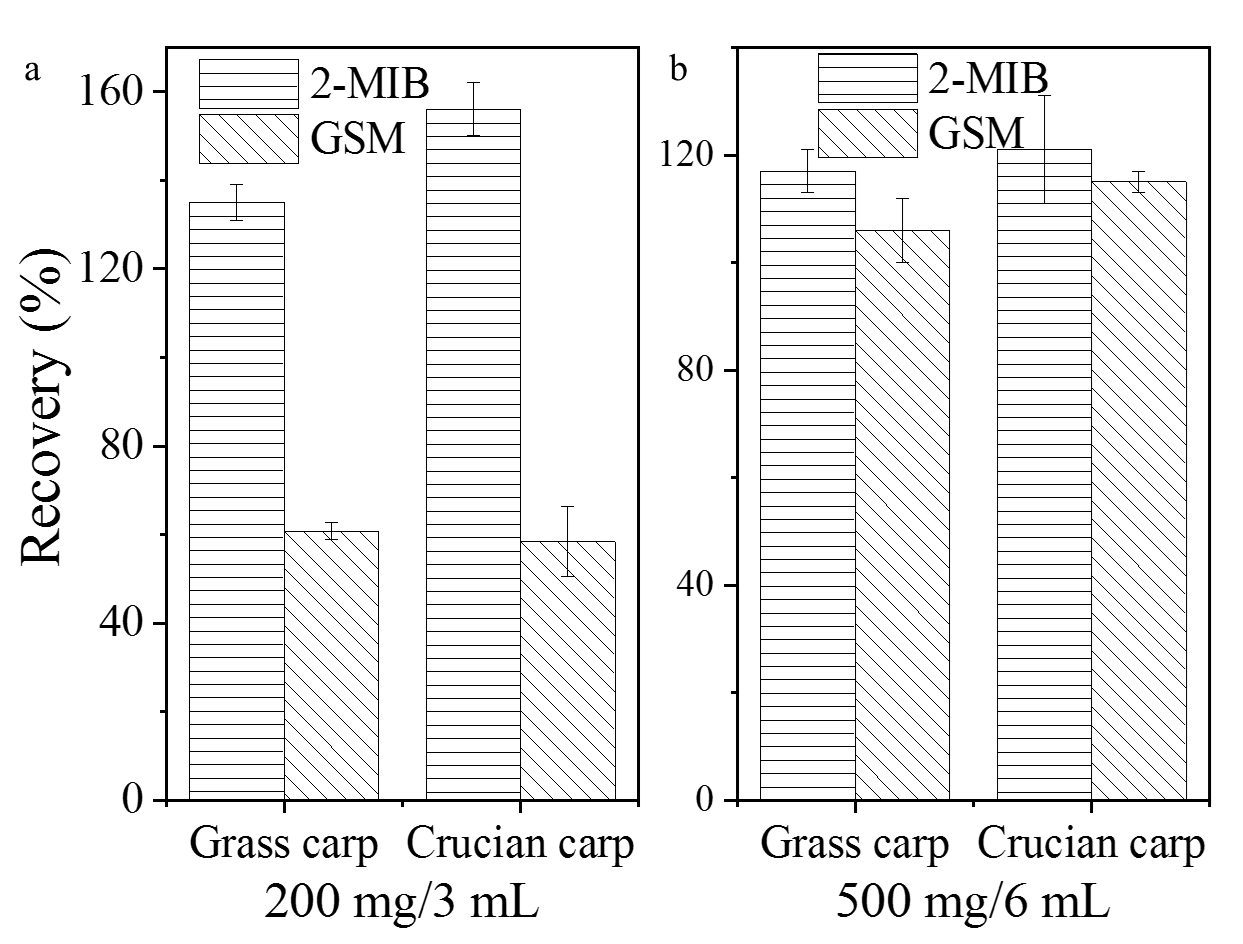


**Figure S**8. Recoveries of 2-MIB and GSM on different silica cartridges (200 mg/3mL a, and 500 mg/3mL, b) in the practical spiked extract.

**Table S**3. Recoveries under different spiking levels in the grass carp.

| spiking level（μg/kg） | recoveries of 2-MIB  (%) | average recovery  (%) | RSD  (%) | recoveries of GSM  (%) | average recovery  (%) | RSD  (%) |
| --- | --- | --- | --- | --- | --- | --- |
| 1.0 | 102/104/97.5/92.2/87.1/94.7 | 96.3 | 6.53 | 90.2/86.0/84.2/80.8/81.4/87.7 | 85.1 | 4.29 |
| 2.0 | 91.5/94.2/85.5/92.4/82.1/90.3 | 89.3 | 5.15 | 86.0/84.4/90.4/82.7/83.5/82.6 | 84.9 | 3.48 |
| 10.0 | 78.6/82.0/84.6/80.8/90.1/82.7 | 83.1 | 4.76 | 90.8/90.3/84.4/82.7/81.4/86.7 | 86.1 | 4.55 |
| 20.0 | 84.2/87.5/94.5/82.1/85.6/81.2 | 85.9 | 5.61 | 90.6/94.6/86.5/84.7/91.2/82.4 | 88.3 | 5.17 |

**Table S**4. Recoveries under different spiking levels in the crucian carp.

| spiking level（μg/kg） | recoveries of 2-MIB  (%) | average recovery  (%) | RSD  (%) | recoveries of GSM  (%) | average recovery  (%) | RSD  (%) |
| --- | --- | --- | --- | --- | --- | --- |
| 2.0 | 88.4/85.4/79.5/84.3/83.2/93.5 | 85.7 | 5.59 | 89.7/85.7/90.6/89.6/83.5/86.4 | 87.6 | 3.19 |
| 10.0 | 85.4/93.6/89.0/84.3/86.5/97.1 | 89.3 | 5.68 | 85.9/90.3/82.5/88.9/84.5/92.2 | 87.4 | 4.23 |
| 20.0 | 80.2/92.5/87.0/81.6/92.9/85.8 | 86.7 | 6.15 | 78.7/86.8/84.8/72.3/85.3/81.3 | 81.5 | 6.63 |

**References**

1. Alghanmi, H. A.; Fo’ad, M. A.; Al-Taee, M. M., Effect of light and temperature on new cyanobacteria producers for geosmin and 2-methylisoborneol. *J. Appl. Phycol.* **2018**, *30*, 319-328.

2. Lindholm-Lehto, P.; Vielma, J.; Pakkanen, H.; Alén, R., Depuration of geosmin-and 2-methylisoborneol-induced off-flavors in recirculating aquaculture system (RAS) farmed European whitefish Coregonus lavaretus. *Journal of food science and technology* **2019**, *56*, 4585-4594.

3. Salto, K.; Karnura, K.; Kataoka, H., Determination of musty odorants, 2-methylisoborneol and geosmin, in environmental water by headspace solid-phase microextraction and gas chromatography-mass spectrometry. *J. Chromatogr. A* **2008**, *1186*, 434-437.

4. Wright, E.; Daurie, H.; Gagnon, G. A., Development and validation of an SPE-GC-MS/MS taste and odour method for analysis in surface water. *Int. J. Environ. Anal. Chem.* **2014**, *94*, 1302-1316.
